# Intraoperative Platelet-Rich Plasma Application Improves Scar Healing After Cesarean Section: A Prospective Observational Pilot Study

**DOI:** 10.3390/healthcare13222905

**Published:** 2025-11-14

**Authors:** Ana-Maria Brezeanu, Dragoș Brezeanu, Simona Stase, Sergiu Chirila, Vlad-Iustin Tica

**Affiliations:** 1 6th Department, Faculty of Medicine, Ovidius University of Constanta, 900527 Constanta, Romania; anmariataras@gmail.com (A.-M.B.); sergiu.chirila@univ-ovidius.ro (S.C.); vtica@eeirh.org (V.-I.T.); 2 County Clinical Emergency Hospital “Sf. Ap. Andrei”, 900591 Constanta, Romania; stase.simona.10d@gmail.com

**Keywords:** platelet-rich plasma, cesarean section, wound healing, postoperative scar, REEDA, POSAS, hematological parameters

## Abstract

**Background:** Cesarean delivery is a frequent surgical procedure associated with postoperative scarring, which may impact both the aesthetic and functional recovery of patients. Platelet-rich plasma (PRP), a concentration of autologous platelets containing bioactive growth factors, has shown promising effects in promoting wound healing and tissue regeneration. However, its efficacy in enhancing cesarean wound healing remains underexplored. **Methods:** This single-arm prospective observational study included 50 patients undergoing cesarean section who received intraoperative PRP in two stages: 5 mL applied before hysterorrhaphy and 5 mL injected subcutaneously before skin closure. Scars were assessed at 7 and 40 days using six validated scales (Manchester, Patient and Observer Scar Assessment Scale (POSAS), Vancouver, Visual Analog Scale (VAS), Numeric Rating Scale (NRS), and REEDA (Redness, Edema, Ecchymosis, Discharge, Approximation). Hematological parameters (hemoglobin, hematocrit, leukocyte count, and platelet count) were monitored to correlate systemic inflammatory response and healing progression. Statistical analysis included the Wilcoxon signed-rank test and Pearson correlation. **Results:** Mean scar scores improved significantly from 8.88 ± 2.13 at day 7 to 6.46 ± 1.23 at day 40 (*p* < 0.001). Hematological parameters improved between day 7 and day 40, reflecting the expected course of postoperative recovery; these were considered secondary outcomes. Exploratory analyses revealed correlations between hemoglobin and POSAS (*r* = 0.42, *p* < 0.05), leukocyte count and REEDA (*r* = 0.68, *p* < 0.01), and platelet count and POSAS (*r* = −0.48, *p* < 0.05). **Conclusions:** Intraoperative PRP administration significantly enhanced postoperative scar healing after cesarean delivery and was associated with reduced inflammation and improved hematological recovery. These findings support the use of PRP as an adjunctive regenerative therapy in obstetric surgery. Randomized controlled trials are warranted to validate efficacy of the intervention, and moreover long-term outcomes.

## 1. Introduction

Cesarean section is among the most performed surgical procedures in obstetrics, with rising global prevalence in both high- and low-resource settings [1]. While the World Health Organization recommends an optimal cesarean rate between 10–15% [2], actual rates often exceed this threshold, reaching up to 19% in Europe and over 30% in some countries [3,4]. Postoperative scarring following cesarean delivery is not only a frequent physical consequence but also a potential source of psychological distress, especially when aesthetic outcomes are suboptimal [5,6].

Surgical wound healing is a dynamic, overlapping process comprising four sequential phases: hemostasis, inflammation, proliferation, and remodeling/maturation [7]. Hemostasis (minutes–hours) involves vasoconstriction and clot formation, providing a fibrin scaffold. Inflammation (hours–days) recruits neutrophils and monocytes/macrophages that clear debris and orchestrate repair signaling [8]. The proliferative phase (days–weeks) is characterized by fibroblast migration, extracellular matrix deposition (notably collagen III), epithelialization, and angiogenesis. Finally, remodeling/maturation (weeks–months) features matrix turnover with increased collagen I content and reorganization that determines the long-term biomechanical and aesthetic properties of the scar [9]. Disruptions in these phases can lead to excessive scarring or delayed healing [10]. Strategies to enhance wound healing and reduce inflammation are of increasing interest in modern obstetric practice. One such approach is the use of platelet-rich plasma (PRP)—an autologous concentration of platelets and growth factors that stimulate tissue regeneration [11,12]. PRP has been widely used in regenerative medicine, including dermatology, orthopedics, and plastic surgery, due to its potential to accelerate epithelialization, reduce local inflammation, and enhance extracellular matrix deposition [13,14,15].

Although PRP has shown promise in managing chronic and surgical wounds, limited data exist regarding its application in obstetric surgery, particularly during cesarean delivery. Preliminary studies suggest that intraoperative PRP may improve scar quality and reduce postoperative discomfort [16,17]. However, the evidence remains inconclusive due to small sample sizes, heterogeneous methodologies, and lack of standardization in PRP preparation and application protocols [18,19,20].

This study aimed to evaluate the efficacy of intraoperative PRP application in improving postoperative scar healing following cesarean section. The investigation included both clinical and hematological parameters, using six validated scar assessment scales (Manchester, Patient and Observer Scar Assessment Scale (POSAS), Vancouver, Visual Analog Scale (VAS), Numeric Rating Scale (NRS), and REEDA) and serial measurements of hemoglobin, hematocrit, leukocytes, and platelets. The study further sought to identify correlations between biological markers and scar evolution, providing a more integrated understanding of the regenerative effects of PRP in obstetric surgery.

## 2. Materials and Methods

### 2.1. Study Design and Ethical Approval

This single-arm prospective observational study was conducted at the County Clinical Emergency Hospital “Sf. Ap. Andrei” and approved by the Ethics Committees of both the hospital and Ovidius University of Constanța (Approval No. 1689/15.12.2021). The protocol was registered at ClinicalTrials.gov (Identifier: NCT06978010). Written informed consent was obtained from all participants prior to inclusion, in accordance with the Declaration of Helsinki. As this was an exploratory pilot study, the sample size (*n* = 50) was chosen based on feasibility within the recruitment period and consistency with prior pilot studies on PRP in obstetric surgery, rather than on a formal power calculation [21].

### 2.2. Patient Selection

A total of 50 patients undergoing elective cesarean section between January and May 2022 were enrolled. Inclusion criteria comprised age between 18 and 40 years, singleton pregnancy, and absence of obstetric or systemic complications. Exclusion criteria included anemia (hemoglobin < 10 g/dL), coagulopathies, autoimmune disorders, local infections, and history of keloid scarring. Patients with a personal history of keloid or hypertrophic scarring were excluded to avoid confounding, as this subgroup follows different biological healing trajectories and may require tailored protocols.

### 2.3. PRP Preparation and Administration

**Figure 1:**
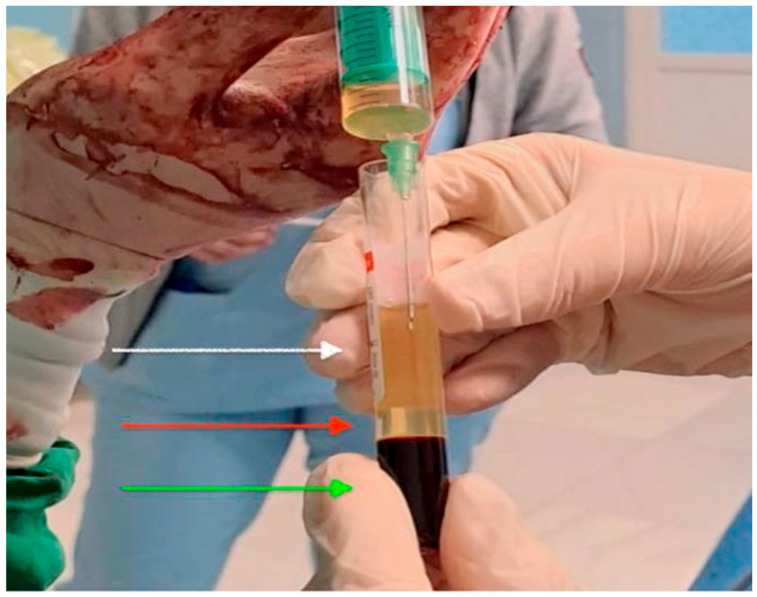
Intraoperative image of platelet-rich plasma prepared for application. There are the three layers seen in the tube: the upper layer of platelet-poor plasma (white arrow), the middle layer of platelet-rich plasma (red arrow) and the bottom layer is the red blood cells (green arrow).

Platelet-rich plasma (PRP) was prepared intraoperatively from autologous blood samples without the use of a commercial kit. A total of 20 mL of peripheral venous blood was collected into sterile tubes containing 3.2% sodium citrate as anticoagulant. Samples were processed using the XC Spin Plus centrifuge (XCmed, Bucharest, Romania) following a two-step protocol: first centrifugation (“soft spin”) at 1600 rpm for 10 min to separate plasma and buffy coat from red blood cells, and second centrifugation (“hard spin”) at 3500 rpm for 7 min to concentrate platelets. The upper platelet-poor plasma (PPP) fraction was discarded, and the platelet pellet was resuspended in the remaining plasma, resulting in a final PRP volume of approximately 6–8 mL as shown in Figure 1. PRP was applied immediately to avoid degradation of growth factors. Administration was performed in two stages: 5 mL infiltrated along the uterine incision margins before hysterorrhaphy, and 5 mL injected subcutaneously prior to skin closure. PRP was autologous, prepared in a sterile field with citrate anticoagulation and without exogenous thrombin, and applied locally during abdominal wall closure after neonatal delivery and uterine repair. Participants were monitored for wound infection, hematoma/seroma, dehiscence, allergic reactions, and readmission. All procedures were carried out by the same surgical team to ensure protocol consistency and reproducibility.

### 2.4. Hematological Parameters

Venous blood samples were collected on postoperative days 7 and 40. The following parameters were analyzed:

**Hemoglobin (g/dL)** and **Hematocrit (%)**: indicators of oxygen transport capacity and postoperative recovery.

**Leukocyte count (/dL)**: marker of systemic inflammation.

**Platelet count (/dL)**: relevant for evaluating regenerative potential.

**ABO blood group and Rh status** were recorded for potential influence on healing, though no stratification was applied.

### 2.5. Scar Assessment Scales

Scar evaluation was performed at **postoperative day 7 and day 40** using six validated clinical tools:

1. **Manchester Scar Scale (MSS)**—clinical assessment of scar thickness, texture, and pigmentation [22].
2. **Patient and Observer Scar Assessment Scale (POSAS)**—incorporates both observer and patient perspectives [23].
3. **Vancouver Scar Scale (VSS)**—evaluates vascularity, pigmentation, pliability, and height [24].
4. **Visual Analog Scale (VAS)**—patient-rated pain intensity (0–10) [25].
5. **Numeric Rating Scale (NRS)**—numerical pain rating (0–10) [26].
6. **REEDA Scale** (Redness, Edema, Ecchymosis, Discharge, Approximation)—evaluates erythema, edema, ecchymosis, discharge, and approximation (0–15 scale) [27].

### 2.6. Statistical Analysis

Data were analyzed using IBM SPSS Statistics version 26. Continuous variables were expressed as means ± standard deviations (SD). The Wilcoxon signed-rank test was applied to compare scar and hematological parameters between day 7 and day 40, considering non-parametric distributions. The Pearson correlation coefficient was used to explore associations between blood parameters and scar scores. Statistical significance was set at *p* < 0.05.

### 2.7. Timing Rationale

We selected two postoperative time points: Postoperative day 7 and Postoperative day 40. The Postoperative day 7 visit aligns with the recommended early postpartum contact (WHO/ACOG) and with common incision checks/suture or staple management in the first 1–2 weeks; this window captures the end of the inflammatory phase and onset of proliferation, when erythema, edema, discharge, and approximation (REEDA components) are most informative. The Postoperative day 40 visit (~6 weeks postpartum) coincides with the standard comprehensive postpartum review, overlapping with early remodeling/maturation of the wound and enabling a clinically relevant appraisal of scar quality and pain.

## 3. Results

### 3.1. Patient Characteristics

A total of 50 women undergoing elective cesarean section were included in the study. The mean maternal age was 27.9 ± 6.1 years (range 18–40), and the mean body mass index (BMI) was 27.9 ± 4.5 kg/m^2^ (range 19.4–42.1). Most patients were primiparous (54%), while 14 women (27%) had at least one previous cesarean delivery and 10 women (19%) reported a history of vaginal birth. These baseline demographic and clinical characteristics are summarized in Table 1.

### 3.2. Scar Healing Outcomes

**Figure 2:**
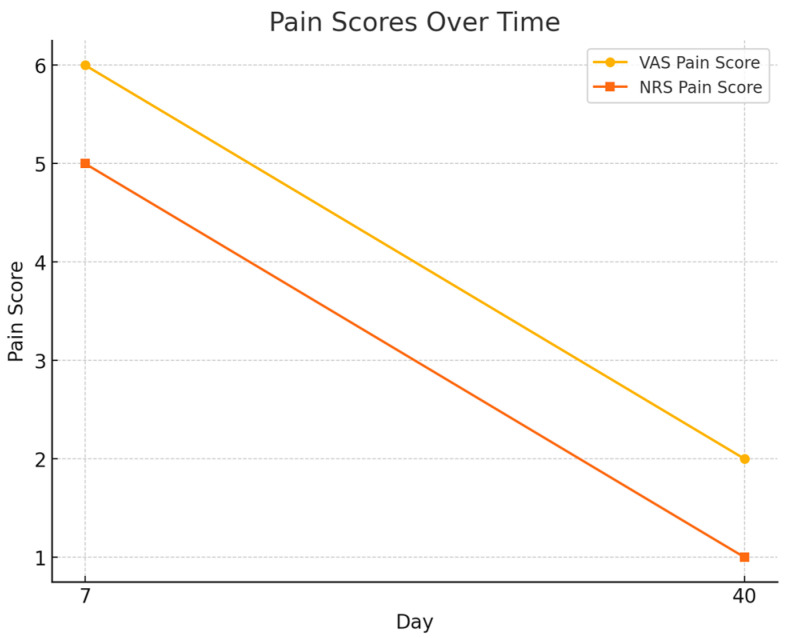
Pain Scores: Visual representation of decreasing VAS and NRS pain scores, proving effective pain management.

A total of 50 patients completed the study protocol with full follow-up at days 7 and 40. The mean overall scar score (based on the combined assessment tools) significantly decreased from 8.88 ± 2.13 at day 7 to 6.46 ± 1.23 at day 40 (*p* < 0.001, Wilcoxon test), indicating improved healing across all evaluated parameters as shown in Table 2 and Figure 2.

Pain intensity also decreased markedly:

VAS score decreased from 6.0 ± 1.5 at day 7 to 2.0 ± 0.9 at day 40.
NRS score declined from 5.0 ± 1.4 to 1.0 ± 0.8, both changes statistically significant (*p* < 0.001).

As shown in Table 3, improvements were also observed consistently across all the other four scar assessment scales between day 7 and day 40. The POSAS score decreased from 32.5 ± 6.0 to 21.4 ± 4.3 (*p* < 0.001), proving marked improvement in both patient-reported and observer-rated scar quality. The REEDA score showed a sharp decline from 7.8 ± 2.0 to 3.4 ± 1.1 (*p* < 0.001), reflecting significant resolution of erythema, edema, and discharge. Similarly, the Vancouver Scar Scale decreased from 8.1 ± 2.1 to 5.2 ± 1.4 (*p* < 0.01), showing enhanced vascularity and pliability of scars. The Manchester Scar Scale also improved (9.2 ± 1.8 to 6.7 ± 1.5, *p* < 0.001), confirming a favorable trajectory in scar morphology.

### 3.3. Hematological Dynamics

**Figure 3:**
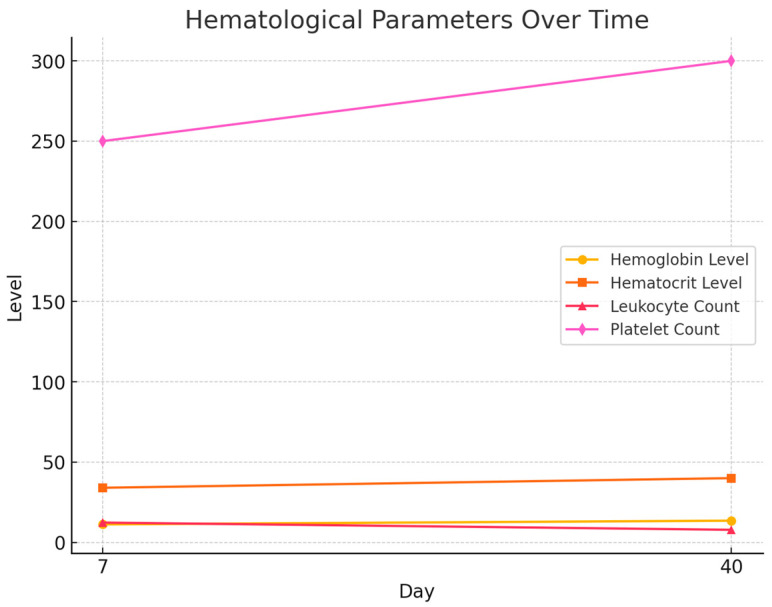
Hematological Parameters at 7 and 40 days. Trends in hemoglobin, hematocrit, leukocyte, and platelet levels over time, indicating recovery and healing.

Hematological parameters were evaluated as secondary outcomes, reflecting overall postoperative recovery rather than PRP-specific effects. As expected, hemoglobin and hematocrit increased by day 40, consistent with recovery from perioperative blood loss, while leukocyte counts decreased in line with resolution of inflammation. Platelet counts increased modestly, and exploratory correlations with scar outcomes are reported below. Table 4 and Figure 3 summarizes the hematological parameters at both timepoints:

Hemoglobin and hematocrit increased significantly by day 40 (*p* < 0.01), consistent with recovery from perioperative blood loss.

Leukocyte counts decreased significantly (*p* < 0.01), suggesting a resolution of acute postoperative inflammation.

Platelet counts increased modestly by day 40 (*p* = 0.04), potentially contributing to tissue regeneration.

**Figure 4:**
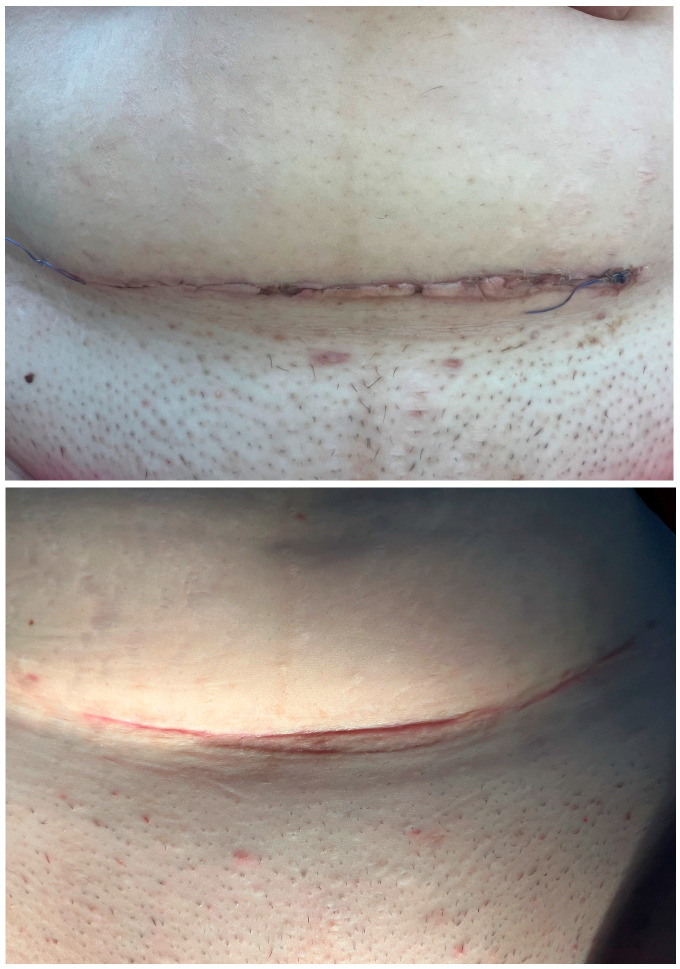
Representative clinical images. Improvement in scar appearance between day 7 and day 40 after intraoperative PRP application.

Clinical photographs were included to provide visual confirmation of scar evolution, complementing the quantitative assessment scales as shown in Figure 4.

### 3.4. Correlation Analyses

Statistically significant correlations were found between biological markers and scar assessment scores:

Hemoglobin and POSAS: *r* = 0.42, *p* < 0.05—suggesting better scar appearance with improved oxygenation.

Leukocytes and REEDA: *r* = 0.68, *p* < 0.01—indicating inflammatory resolution mirrored by leukocyte decline.

Platelets and POSAS: *r* = −0.48, *p* < 0.05—suggesting enhanced tissue regeneration with higher platelet levels.

## 4. Discussion

This study evaluated the effects of intraoperative platelet-rich plasma (PRP) administration on postoperative scar healing following cesarean delivery. The platelet concentration achieved with this method is generally about 4–5 times higher than baseline whole blood levels, as described in previous studies [12,28,29]. The findings demonstrate a statistically significant improvement in scar appearance and pain reduction by day 40, supported by both clinical scales and hematological markers. These results are consistent with previous studies highlighting PRP’s regenerative properties and its capacity to accelerate tissue repair through the release of growth factors such as PDGF, TGF-β, VEGF, and EGF [12,13,30].

The choice of postoperative day 7 and postoperative day 40 was pragmatic and evidence-informed: postoperative day 7 aligns with routine early postpartum contact (7–14 days), when most early wound issues declare themselves, and postoperative day approximates the ~6-week comprehensive postpartum visit [31,32]. Biologically, these checkpoints straddle the transition from inflammation into proliferation (postoperative day 7) and the onset of remodeling (postoperative day 40), offering complementary views of scar dynamics and patient-reported pain. We did not include a “baseline” assessment at day 0 because validated scar scales (POSAS observer, Vancouver, REEDA) are not designed for an intraoperative or immediate-post closure environment and would be confounded by anesthesia, dressings, and strict sterility requirements and also immediate post-op measurements are dominated by acute surgical factors such as hemodilution, fluids or analgesia rather than biologically meaningful scar features [7,8].

The decrease in overall scar scores, particularly in the POSAS, Vancouver, and REEDA scales, aligns with published data indicating that PRP can enhance neovascularization, modulate inflammation, and improve collagen remodeling [16,19,32]. Notably, the pain-related scores (VAS and NRS) decreased substantially, suggesting that PRP may also contribute to neurosensory modulation and nociceptive suppression in healing tissues. These effects have been previously described in studies involving PRP use for surgical incisions and chronic wounds [15,20].

The hematological dynamics further support the observed clinical improvements [33]. The increase in hemoglobin and hematocrit levels by day 40 reflects overall systemic recovery and adequate tissue perfusion. Similarly, Chaichian et al. reported improved scar aesthetics in association with perioperative hemoglobin recovery [15]. Our findings align with previous reports. Tehranian et al. observed that reductions in leukocyte count paralleled lower REEDA scores in high-risk cesarean patients treated with PRP [16]. The negative correlation between platelet counts and POSAS scores suggests a potential dose–response relationship, whereby higher platelet concentrations were associated with lower POSAS values, reflecting improved scar morphology, vascularity, pliability, and patient-reported satisfaction, as also reported in studies by Barwijuk et al. [34] and Tehranian et al. [16]. It should be emphasized that hematological improvements are expected in the natural course of postpartum recovery and should therefore be regarded as secondary, exploratory findings. Their main value lies in the observed correlations with scar scores, rather than in suggesting a direct causal effect of PRP. Although ABO blood group and Rh status were recorded, these parameters were not stratified in our analysis. Their inclusion was based on prior suggestions that blood group antigens may influence hemostasis, inflammation, and tissue repair [35]. In our cohort, no association between ABO/Rh status and scar outcomes was observed, which may be due to the limited sample size. Future studies with larger populations could explore whether genetic or immunohematological factors contribute to variability in cesarean wound healing.

Our results are in concordance with a randomized study by Chaichian et al. [15], which demonstrated improved scar aesthetics and pain perception in patients receiving PRP after cesarean delivery. Similarly, Elkhouly et al. observed faster epithelialization and reduced REEDA scores in high-risk obstetric patients treated with PRP. These findings support the biological plausibility of PRP-mediated regeneration through enhanced fibroblast activity, angiogenesis, and extracellular matrix deposition.

Unlike orthopedic or plastic surgery incisions, cesarean scars evolve under systemic postpartum physiological changes and are directly linked to maternal reproductive health and psychosocial recovery [36,37]. Cesarean wound healing differs from other cutaneous incisions because it occurs in a hormonally dynamic and highly vascularized postpartum environment, subject to unique mechanical stressors from uterine involution and abdominal wall tension. Moreover, cesarean scars carry specific psychological implications for maternal well-being and body image, underscoring the clinical relevance of improving scar quality in this population [38,39]. This contextual difference underscores the novelty of our study, which evaluates intraoperative PRP specifically in obstetric surgery, bridging regenerative medicine with maternal care.

Evidence from clinical trials indicates that intraoperative autologous PRP is well-tolerated, with improved scar outcomes and no increase in short-term complications [40]. This aligns with broader wound-care meta-analyses showing no excess infections or adverse events versus standard care [41]. Prior randomized and single-blind cesarean studies reported improved healing with no PRP-related safety signal (including no dehiscence in either arm in the single-blind trial; and no adverse events in the PRP arm in the RCT) [16,34]. While rare adverse events have been described in other fields of PRP use, we observed no PRP-related adverse events in our cohort.

However, several limitations must be acknowledged. The main limitation of the present study is the absence of a control group. Without comparison to patients receiving standard care, it is not possible to fully distinguish the regenerative effect of PRP from the natural trajectory of wound healing. Although the significant improvements observed across all scar assessment scales are encouraging, these results must be interpreted with caution. Future randomized controlled trials with placebo or standard-care arms are required to confirm the causal relationship between intraoperative PRP administration and improved scar healing after cesarean section. We considered the use of pre-existing or historical data as a comparator; however, heterogeneity in surgical techniques, follow-up schedules, and outcome measures would have introduced significant bias. Moreover, validated scar scales such as POSAS or REEDA are not consistently reported in retrospective series. For these reasons, we deliberately refrained from including external datasets and instead present this study as an observational pilot. This approach avoids misleading comparisons and provides a foundation for future randomized controlled trials with contemporaneous control groups.

Another limitation is the absence of blinding. Because all patients received PRP, neither the surgical team nor the patients were blinded to the intervention. This may have introduced performance and expectation bias, especially in patient-reported outcomes. Future randomized controlled trials with sham or placebo interventions are needed to mitigate this risk.

Another limitation concerns the absence of PRP stratification by type (leukocyte-rich vs. leukocyte-poor), which may influence inflammatory and regenerative responses differently [42,43]. Standardization of PRP preparation and classification (e.g., using the PAW or DEPA systems) remains a challenge across clinical studies and should be addressed in future research.

Finally, no a priori power calculation was performed, as the study was intended to provide preliminary, hypothesis-generating data to inform the design of future randomized controlled trials.

### 4.1. Originality and Innovative Contributions

This study offers an original contribution to the field of regenerative medicine in obstetrics by systematically evaluating the intraoperative application of autologous platelet-rich plasma (PRP) during cesarean section and its impact on postoperative wound healing. Unlike most studies that focus solely on clinical outcomes, our research integrates both visual scar assessment using validated scales and biological monitoring through hematological parameters.

The originality of the study lies in several key aspects:

1. Implementation of a dual-stage intraoperative PRP administration protocol, targeting both the myometrial and subcutaneous layers.
2. Use of six validated clinical scales (Manchester, POSAS, Vancouver, VAS, NRS, REEDA) to comprehensively assess scar morphology, vascularity, and patient-reported outcomes.
3. Analysis of objective hematological markers (hemoglobin, hematocrit, leukocytes, platelets) in relation to scar evolution, creating a unique link between systemic and local healing responses.
4. Registration of the clinical protocol in an international database (ClinicalTrials.gov: NCT06978010), ensuring methodological transparency and ethical compliance.

By bridging regenerative therapy with surgical practice, this research promotes a translational approach and lays the foundation for integrating PRP into standardized postoperative care protocols aimed at enhancing cesarean wound healing.

### 4.2. Future Research Directions and Clinical Applicability

Building on the positive findings of this pilot study, future research should aim to:

1. Conduct larger randomized controlled trials (RCTs) with placebo or standard-care control arms to confirm causality and minimize bias.
2. Evaluate long-term outcomes of scar remodeling, including elasticity, function, and the risk of hypertrophic or keloid scar formation.
3. Standardize PRP preparation techniques and classify its biological profile (e.g., leukocyte-rich vs. leukocyte-poor), in line with PAW or DEPA classification systems.
4. Explore the use of PRP in other gynecologic surgeries, such as myomectomy, hysterectomy, or endometriosis-related procedures, with tailored indications.
5. Future studies should specifically evaluate women with a predisposition to keloid or hypertrophic scars, as this group may particularly benefit from PRP’s anti-inflammatory and regenerative properties.

In clinical practice, PRP represents a safe, autologous, and easy-to-apply adjunct for improving surgical recovery in obstetrics. Its potential to reduce postoperative pain, enhance scar quality, and accelerate tissue regeneration may translate into improved maternal outcomes and patient satisfaction.

## 5. Conclusions

Intraoperative administration of autologous PRP during cesarean section was associated with significant improvements in scar quality and pain reduction at 40 days postpartum. Hematological changes paralleled scar improvement but should be regarded as secondary outcomes. The absence of a control group and the short follow-up period limit the strength of causal inferences. Randomized controlled trials are warranted to validate efficacy of the intervention, and moreover, long-term outcomes.

## Figures and Tables

**Table 1:** Patient characteristics.

Variable	Value
Age (years)	27.9 ± 6.1 (range 18–40)
BMI (kg/m^2^)	27.9 ± 4.5 (range 19.5–42.1)
Primiparous	26 (54%)
Previous cesarean deliveries	14 (27%)
Previous vaginal deliveries	10 (19%)

**Table 2:** Scar and pain score at 7 and 40 days after cesarean section with intraoperative PRP application.

Day	Scar Score Mean	Std Dev Scar Score	VAS Pain Score	NRS Pain Score
7	8.88	2.13	6	5
40	6.46	1.23	2	1

**Table 3:** Scar assessment scores at day 7 and day 40 after cesarean section with intraoperative PRP application.

Scale	Day 7 (Mean ± SD)	Day 40 (Mean ± SD)	*p*-Value
Manchester Scar Scale (MSS)	9.2 ± 1.8	6.7 ± 1.5	<0.001
Patient and Observer Scar Assessment Scale (POSAS)	32.5 ± 6.0	21.4 ± 4.3	<0.001
Vancouver Scar Scale (VSS)	8.1 ± 2.1	5.2 ± 1.4	<0.01
REEDA Scale (Redness, Edema, Ecchymosis, Discharge, Approximation)	7.8 ± 2.0	3.4 ± 1.1	<0.001

Values are expressed as mean ± standard deviation (SD).

**Table 4:** Hematological parameters at 7 and 40 days.

Day	Hemoglobin (g/dL) Level Mean	Hematocrit (%) Level Mean	Leukocyte (Cells/dL) Count Mean	Platelet (Cells/dL) Count Mean
7	11.2 g/dL	34%	12,300/dL	250.000/dL
40	13.5 g/dL	40%	7800/dL	300.000/dL

## Data Availability

The original contributions presented in this study are included in the article. Further inquiries can be directed to the corresponding author.
